# Pancreatic Insulin-Producing Cells Differentiated from Human Embryonic Stem Cells Correct Hyperglycemia in SCID/NOD Mice, an Animal Model of Diabetes

**DOI:** 10.1371/journal.pone.0102198

**Published:** 2014-07-10

**Authors:** Xiu-feng Hua, Yan-wei Wang, Yu-xiao Tang, Sheng-qiang Yu, Shao-hua Jin, Xiao-mei Meng, Hua-feng Li, Fu-jun Liu, Qiang Sun, Hai-yan Wang, Jian-yuan Li

**Affiliations:** 1 Department of Endocrinology, Yuhuangding Hospital, Yantai, Shandong Province, China; 2 Research Center of Stem Cell Engineering of Shandong, Central Laboratory of Yuhuangding Hospital, Yantai, Shandong Province, China; 3 Department of Urology, Yuhuangding Hospital, Yantai, Shandong Province, China; 4 Department of clinical laboratory, Yuhuangding Hospital, Yantai, Shangdong Province, China; 5 Department of Orthopedics, Chinese people's Liberation Army Navy 407 hospital, Yantai, Shandong Province, China; Children's Hospital Boston/Harvard Medical School, United States of America

## Abstract

**Background:**

Human pancreatic islet transplantation is a prospective curative treatment for diabetes. However, the lack of donor pancreases greatly limits this approach. One approach to overcome the limited supply of donor pancreases is to generate functional islets from human embryonic stem cells (hESCs), a cell line with unlimited proliferative capacity, through rapid directed differentiation. This study investigated whether pancreatic insulin-producing cells (IPCs) differentiated from hESCs could correct hyperglycemia in severe combined immunodeficient (SCID)/non-obese diabetic (NOD) mice, an animal model of diabetes.

**Methods:**

We generated pancreatic IPCs from two hESC lines, YT1 and YT2, using an optimized four-stage differentiation protocol in a chemically defined culture system. Then, about 5–7×10^6^ differentiated cells were transplanted into the epididymal fat pad of SCID/NOD mice (n = 20). The control group were transplanted with undifferentiated hESCs (n = 6). Graft survival and function were assessed using immunohistochemistry, and measuring serum human C-peptide and blood glucose levels.

**Results:**

The pancreatic IPCs were generated by the four-stage differentiation protocol using hESCs. About 17.1% of differentiated cells expressed insulin, as determined by flow cytometry. These cells secreted insulin/C-peptide following glucose stimulation, similarly to adult human islets. Most of these IPCs co-expressed mature β cell-specific markers, including human C-peptide, GLUT2, PDX1, insulin, and glucagon. After implantation into the epididymal fat pad of SCID/NOD mice, the hESC-derived pancreatic IPCs corrected hyperglycemia for ≥8 weeks. None of the animals transplanted with pancreatic IPCs developed tumors during the time. The mean survival of recipients was increased by implanted IPCs as compared to implanted undifferentiated hESCs (*P*<0.0001).

**Conclusions:**

The results of this study confirmed that human terminally differentiated pancreatic IPCs derived from hESCs can correct hyperglycemia in SCID/NOD mice for ≥8 weeks.

## Introduction

The development of a cellular therapy for diabetes requires a renewable source of human insulin-secreting cells that respond to glucose in a physiologic manner. Mature islet transplantation has been proposed as a promising treatment for type 1 diabetes [1], [2]. However, an acute shortage of deceased organ donors currently limits the wider application of islet transplantation. One approach to overcome the limited supply of donor pancreases is to generate IPCs from stem cells with high proliferative and differentiating potential [3]. hESCs have the potential to differentiate into specialized cells of all three primary germ-layers, including pancreatic IPCs [4], [5]. hESCs represent a potentially unlimited source of transplantable islet cells for treating diabetes [6]. For this reason, systematic and mechanistic studies are required to examine the potential for using hESCs as a stem cell-based therapy for type 1 diabetes.

Several groups have reported stepwise protocols for mimicking the development of the pancreas in vivo. D'Amour et al [7] reported a five-stage protocol for differentiating hESCs into pancreatic hormone-expressing endocrine cells that secreted insulin in response to various secretagogues but not to glucose in vitro. Zhang et al [8] reported a four-stage protocol for differentiating hESCs into mature IPCs that secreted insulin/C-peptide in response to glucose stimulation. After comparing the different protocols, we chose a four-stage protocol for inducing the differentiation of hESCs into IPCs, and transplanted the cells into SCID/NOD mice to assess graft survival and function by performing immunohistochemistry, and measuring serum human C-peptide levels and blood glucose levels. We found that these terminally differentiated cells were morphologically and functionally similar to pancreatic islets, and protected mice against streptozotocin (STZ)-induced hyperglycemia.

## Methods

### hESC culture and differentiation

This study was approved by Ethics Committee of The Medical College of Qingdao University, China.

The hESC lines YT1 and YT2 [9] were derived and characterized at our institute. The hESCs were cultured in Dulbecco's modified Eagle's medium (DMEM)/F12 supplemented with 20% KnockOut serum replacement (KSR) and 4 ng/mL of basic fibroblast growth factor (bFGF) on mouse embryonic fibroblast feeders. Colonies of hESCs were digested with 10 mg/mL collagenase IV into small clumps for differentiation. The hESC clumps were replated on Matrigel (BD Biosciences, Franklin Lakes, NJ, USA; 1∶50)-coated dishes to provide coverage of 60%. The cells were incubated with RPMI1640 containing 0.2% fetal bovine serum (FBS), 0.5×N2 and 0.5×B27 supplemented with 100 ng/mL activin A (R&D Systems, Minneapolis, MN, USA) and 1 µM wortmannin for 4 days. The differentiated cells were cultured in RPMI1640 supplemented with 0.5% FBS, 0.5% insulin/transferrin/selenium (ITS), 0.5×B27, 2 µM retinoic acid (RA) (Sigma, St. Louis, MO, USA), 20 ng/ml fibroblast growth factor-7 (FGF-7), and 50 ng/mL Noggin for 4 days. The cells were then incubated for 5 days in high-glucose DMEM supplemented with 0.5% FBS, 1% ITS, 1×N2, and 50 ng/mL epidermal growth factor (EGF) (Sigma). The cells expanded and attained confluency. Finally, the cells were cultured in DMEM/F12 containing 1% ITS, 10 ng/ml bFGF, 10 mM nicotinamide (Sigma), 50 ng/ml exendin-4 (Sigma), and 10 ng/ml bone morphogenetic protein 4 (BMP4) for maturation. All media and supplements were from Invitrogen (Carlsbad, CA, USA) and growth factors were from Peprotech (Rocky Hill, NJ, USA), unless otherwise specified.

### Immunohistochemistry

The induced cells were fixed in 4% paraformaldehyde for 15 min, permeabilized for 10 min in 0.2% Triton X-100/phosphate-buffered saline (PBS), and blocked with 10% serum. The cells were incubated with the primary antibody overnight at 4°C and then with the secondary antibody (fluorescein isothiocyanate [FITC]-conjugated donkey anti-rabbit IgG, Cy3-conjugated donkey anti-guinea pig IgG, or Cy3-conjugated donkey anti-rabbit IgG; Millipore, Billerica, MA, USA) for 60 min at room temperature. The following primary antibodies were used: rabbit anti-PDX1 (1∶1000; Abcam, Cambridge, UK), guinea pig anti-insulin (1∶50; Dako, Glostrup, Denmark), and mouse anti-C-peptide (1∶200; Linco/Millipore). Images were captured with a confocal microscope (Zeiss, Jena, Germany). Immunostaining was performed in samples prepared without the primary antibody as a negative control.

The grafts retrieved from mice were preserved in Bouin's solution for 24 h and embedded in paraffin. Then, 5-µm-thick sections were cut and placed on slides. Immunohistochemistry was performed as previously described [10].

### Flow cytometry

The induced hESCs were washed with PBS and dissociated into single-cell suspensions with 0.25% trypsin-EDTA, and adjusted to 1×10^6^ cells/mL with PBS. The following primary and secondary antibodies were used: anti-insulin (guinea pig IgG; 1∶500; Dako) and anti-C-peptide (mouse IgG; 1∶200; Linco/Millipore), FITC-conjugated donkey anti-rabbit IgG, Cy3-conjugated donkey anti-guinea pig IgG, and Cy3-conjugated donkey anti-rabbit IgG. After being washed, the cells were analyzed by flow cytometry.

### RNA isolation and semi-quantitative RT-PCR

Total RNA was extracted using RNAiso Plus reagent (TaKaRa, Dalian, China) according to the manufacturer's instructions. Then, 2 µg of total RNA was reverse transcribed with ReverTraAce (TOYOBO, Osaka, Japan). Taq polymerase (TaKaRa, Dalian, China) was used for PCR and the reaction conditions comprised initial denaturation at 94°C for 5 min, followed by an appropriate number of cycles of denaturation at 94°C for 30 s, annealing at 60°C for 30 s, and extension at 72°C for 30 s, and a final extension step at 72°C for 10 min. The primer sequences and the length of each product are shown in Table 1.The PCR products were separated on a 1% (w/v) agarose gel, and were visualized by ethidium bromide staining with a 2 kb DNA ladder to estimate the size of the products. Amplification of the housekeeping gene β-actin was used as a positive control. A negative control for amplimer contamination was done using a complete PCR reaction mix.

**Table 1:** Primer sequences, RT-PCR conditions, and product size.

Gene	Primer sequences (forward/reverse)	Annealing temperature	Products size (bp)
Pdx1	ACCAAAGCTCACGCGTGGAAA	60	199
	TGATGTGTCTCTCGGTCAAGTT		
Insulin1	GAGGCCATCAAGCACCATCAC	60	373
	GGCTGCGTCTAGTTGCAGTA		
Hnf4α	ATCAGAAGGCACCAACCTCAAC	60	197
	TGTCTTTGCCACCACGCACT		
Isl-1	ATTTCCCTATGTGTTGGTTGCG	60	229
	CGTTCTTGCTGAAGCCGATG		
Glut2	GCTACCGACAGCCTATTCTA	60	267
	CAAGTCCCACTGACATGAAG		
Nkx6-1	ACACGAGACCCTCTTTTTCCG	60	336
	TGCTGGACTTGTGCTTCTTCAAC		
β-actin	CCTCGAAACTACCTTCAACTC	60	387
	GCCATGCCAATCTCATCTT		

### Measurement of insulin and C-peptide secretion by electrochemiluminescence immunoassays

The levels of human insulin/C-peptide in culture supernatants following glucose stimulation and after ultrasonic cell lysis were measured using Elecsys 1010 insulin and C-peptide electrochemiluminescence immunoassays (Roche, Penzberg, Germany). To test whether the secretion of insulin and C-peptide from differentiated hESCs occurred in a glucose-dependent manner, cells were stimulated with 5.5, 16.7, or 25 mM glucose. Undifferentiated hESCs incubated in the same conditions were used as a control. After pre-incubation with Krebs–Ringer buffer at 37°C for 90 min, the differentiated hESCs were incubated with Krebs–Ringer buffer containing 5.5, 16.7, or 25 mM glucose for 60 min at 37°C. The conditioned supernatants were collected and analyzed. The total protein content was determined using a BCA Protein Assay Kit (PIERCE/Thermo Fisher Scientific, Inc., Rockford, IL, USA).

### STZ-induced diabetic mice

Male SCID/NOD mice aged 6–8 weeks were obtained from Vital River Laboratories, (Beijing, China) were used in this study. The mice were maintained under specific pathogen-free conditions in an animal facility with controlled humidity (55%±5%), light (12/12 h light/dark), and temperature (22°C±1°C). The air in the facility was passed through a HEPA filter system designed to exclude bacteria and viruses. Animals were fed with ad libitum access to a standard irradiated diet. The experimental protocols and animal care procedures were approved by the Ethics Review Board of Qingdao University Medical College.

To induce experimental diabetes, 70 mg/kg STZ (in 0.1 M citrate buffer, pH 4.5) was injected intraperitoneally to mice every day. Four days after STZ injection, we started measuring blood glucose levels every day using tail vein blood with a glucose meter (Roche, Basel, Switzerland). The mean blood glucose of mice was 6.3±2.1 mmol/L before STZ injection. At 1–5 days after STZ administration, all STZ-treated mice exhibited hyperglycemia, with mean blood glucose levels of 9.2±3.6, 13.6±5.9, 16.9±6.4, 23.9±6.8, and 24.6±10.3 mmol/L, on days 1–5, respectively. Three days after STZ treatment, the mean blood glucose levels exceeded 16.7 mmol/L in all mice. Once the blood glucose levels stabilized and exceeded 16.7 mmol/L for ≥3 consecutive days after STZ administration, they were considered diabetic. These diabetic mice were then injected with long-acting insulin every day until transplantation. The unsuccessful induced diabetic mice were excluded from the study. Mice were monitored for body weight and blood glucose levels every day or weekly.

### Graft preparation and transplantation

Confluent differentiated cells were detached from the culture plates and were cut into appropriately sized cell clusters using a cell scraper. The aggregates were centrifuged, and the pellet was resuspended in media to a final volume of 500 µL. Before surgery, the mice were anesthetized by using Nembutal (intraperitoneal injection, 50 mg/kg).We transplanted the differentiated hESCs into the epididymal fat pads of male SCID/NOD mice as previously described [11]. A total of 20 mice were transplanted with hESC-derived IPCs, and 6 mice were transplanted with undifferentiated hESCs. Mice were euthanized using carbon dioxide when deemed unhealthy. Each mouse received 5–7×10^6^ hESCs. After recovery, the buprenorphine was injected subcutaneously at a dosage of 0.75 mg/kg to relive pain. Eight hours later, the buprenorphine was injected repeatedly. Engrafted epididymal fat pads were fixed in Bouin's solution. After 8 weeks of transplantation, the experimental mice were sacrificed with carbon dioxide and then the grafts from experimental mice were collected and analyzed.

### C-peptide measurement in serum

Graft survival was assessed by serum measurements of human C-peptide levels. C-peptide was measured using electrochemiluminescence immunoassays (Roche, Penzberg, Germany).The assay was performed as described by the manufacturer.

### Histological analysis of graft development in vivo

To assess the differentiation potential of the hESCs, we grafted the differentiated cells into the epididymal fat pad of 20 SCID/NOD mice. Aggregates of 5–7×10^6^ cells were deposited onto a Gelfoam sponge and overlaid with Matrigel. Mice were anaesthetized with inhalable isofluance and the prepared grafts were implanted into the epididymal fat pad in each male SCID/NOD mouse. All mice were treated with oral enrofloxacin (Bayer Animal Health) for 1 week(100 µg/mL in drinking water) after implantation. Eight weeks after implantation, the grafts were retrieved from the epididymal fat pad and subjected to immunohistological analysis.

### Statistical analysis

The percentages of insulin- or C-peptide-positive cells were assessed in three independent experiments. Cells were also stained with 4′,6-diamidino-2-phenylindole to estimate the total number of cells. All data are presented as the mean ± standard error unless otherwise stated. Results were analyzed by unpaired *t*-tests and one-way analysis of variance to detect significant differences among groups. Kaplan–Meier survival curves and the log-rank test were used to assess survival of SCID/NOD mice. Values of *P*<0.05 were considered statistically significant. Statistical tests were performed using SPSS software (SPSS Inc., Chicago, IL, USA).

## Results

### Pancreatic differentiation of hESCs

**Figure 1:**
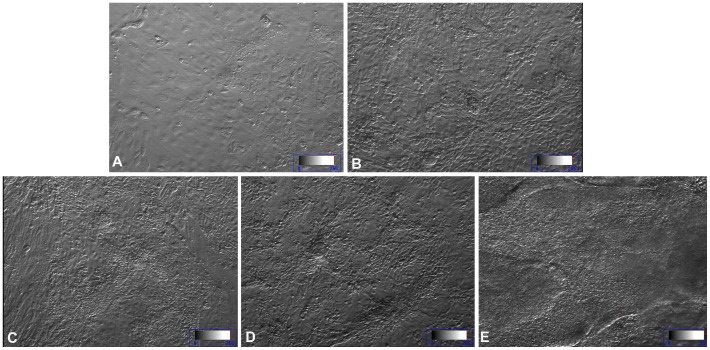
Differentiation of hESCs into a pancreatic lineage in a four-stage protocol. (A) hESC colonies were passaged onto 1% Matrigel-coated dishes for attachment. (B) In stage 1, the hESC cluster was incubated in RPMI1640 with 0.2% FBS, 0.5×N2, 0.5×B27, 100 ng/mL activin A, and 1 µM wortmannin. (C) In stage 2, cells were culturing in RPMI1640 with 0.5% FBS, 0.5% ITS, 0.5×B27, 2 µM RA, 20 ng/mL FGF7, and 50 ng/mL Noggin. (D) In stage 3, cells were cultured in high-glucose DMEM supplemented with 0.5% FBS, 1% ITS, 1×N2, and 50 ng/mL EGF. (E) In stage 4, the cells were cultured in DMEM/F12 with 1% ITS, 10 ng/mL bFGF, 10 mM nicotinamide, 50 ng/mL exendin-4, and 10 ng/mL BMP4 for maturation. Scale bars: 100 µm.

Inverted microscopy confirmed that the undifferentiated hESCs proliferated through clonal growth. The hESCs were then cultured using a four-stage in vitro differentiation protocol [8] with some modifications (Figure 1). Initially, the hESCs were induced to form definitive endodermal cells. Then, the cells were differentiated into pancreatic endodermal cells, and finally to insulin-producing cells.

Adherent cultures were differentiated under feeder-free conditions in the absence of FBS. The hESC colonies were plated on 1% Matrigel for 3 days in medium. On Day 8 of culture, the cells began to bounce back and were characterized by a smaller volume with circular shapes.

### Immunohistochemistry analysis

**Figure 2:**
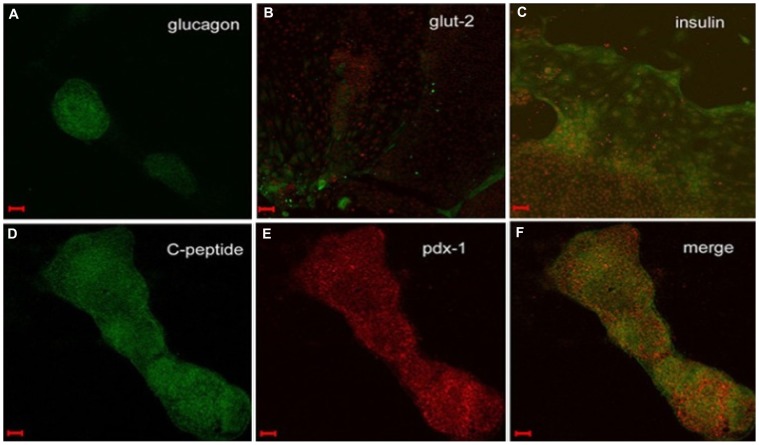
Immunofluorescence analysis of differentiated hESCs at all stages. (A) At Day 14, the differentiated hESCs were positively stained with antibodies against glucagon. (B) The mature differentiated hESCs expressed GLUT-2 (green). The red color represents cell nuclei stained with propidium iodide. (C–E) At Day 20, the differentiated hESCs were positively stained with antibodies against insulin (C; green), C-peptide (D; green), and PDX-1 (E; red). (F) Merged image of D and E showing colocalization of C-peptide (green) and PDX-1 (red). Scale bars: 100 µm.

As shown in Figure 2, immunohistochemistry confirmed that the differentiated hESCs expressed pancreatic markers, including insulin, glucagon, PDX-1, and C-peptide.

### RT-PCR analysis

**Figure 3:**
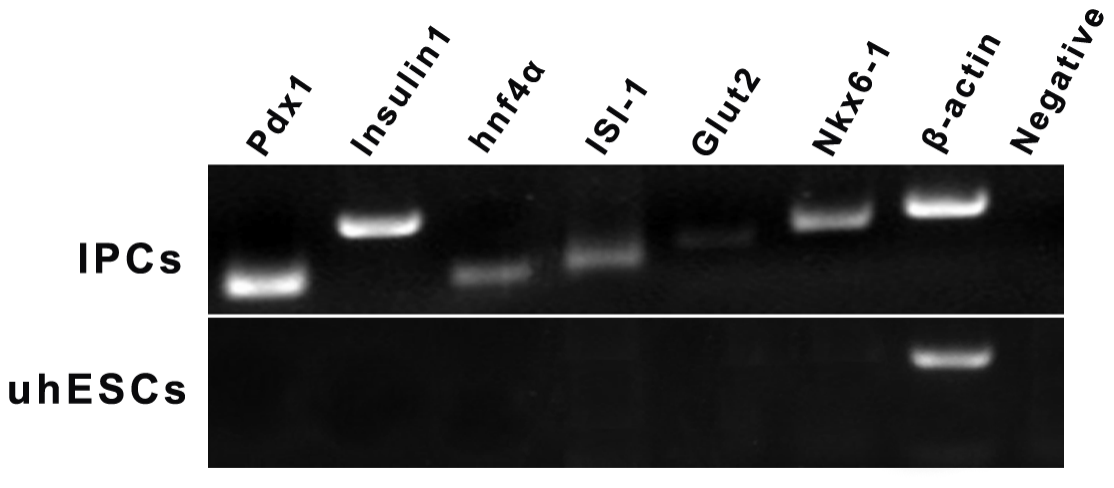
RT-PCR analysis of pancreatic islet cell-specific markers. In contrast to undifferentiated hESCs (uhESCs), Pdx1, Insulin-1, hnf4α, Isl-1, Glut2, and Nkx6-1 were detected in hESC-derived IPCs.

RT-PCR analyses confirmed that the differentiated hESCs expressed Pdx1, Insulin-1, Hnf4α, Isl-1, Glut2, and Nkx6-1 (Figure 3).

### Flow cytometry

**Figure 4:**
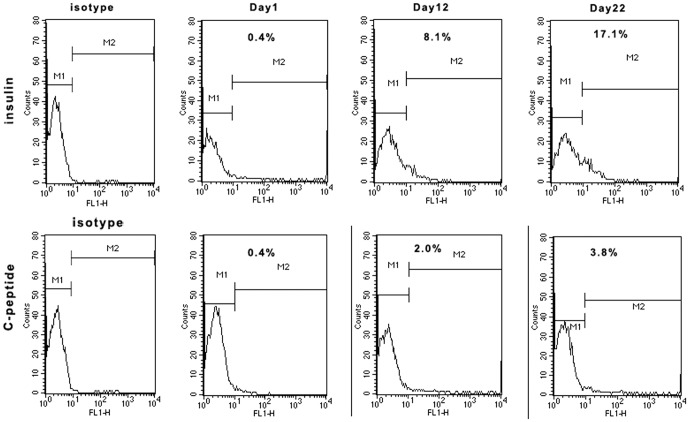
Flow cytometry analysis of the percentages of cells expressing insulin and C-peptide on Days 1, 12 and 22.

The percentages of cells expressing human C-peptide and insulin were determined on Days 1, 12, and 22 by flow cytometry. On day 1, 0.4% of the cells were positive for insulin and C-peptide. After 12 day's induction, 7.1% and 2.0% of the cells were positive for insulin and C-peptide, respectively. On Day 22, >17.1% and >3.8% of differentiated hESCs were positive for insulin and C-peptide, respectively (Figure 4).

### Secretion of C-peptide and insulin from differentiated hESCs

Insulin could not be detected in the supernatant of cells after stage 1 of the differentiation protocol. However, hESCs at stages 2 and 3 of induction are capable of secreting insulin. Mature cells obtained after stage 4 (10^5^ cells/well) were preincubated with Krebs–Ringer buffer containing 5.5, 16.7, or 25 mM glucose for 60 min at 37°C; the insulin levels in the supernatant of these batches of cells were 112.8±30.6, 176.6±40.6, and 185.0±43.7 µU/L, respectively. C-peptide secretion were (8.6±2.7)ng/mL, (12.3±4.9) ng/mL, (15.6±4.9) ng/mL, respectively.

### Transplantation of pancreatic cells into the epididymal fat pads of diabetic mice

**Figure 5:**
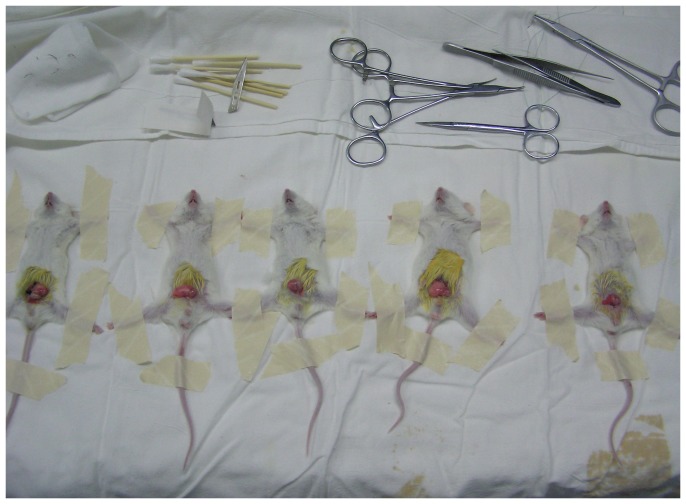
Pancreatic IPCs derived from hESCs were transplanted into the epididymal fat pad of diabetic immunoincompetent mice.

**Figure 6:**
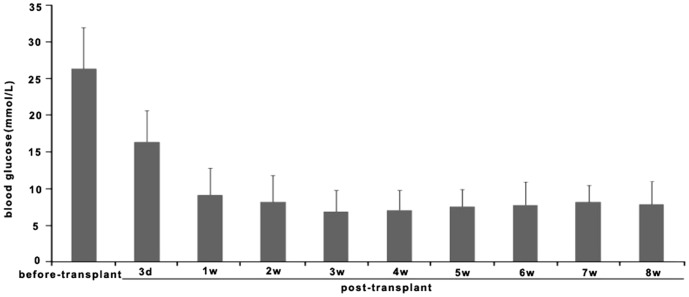
Blood glucose levels measured at the indicated times after transplanting pancreatic IPCs into diabetic mice.

**Figure 7:**
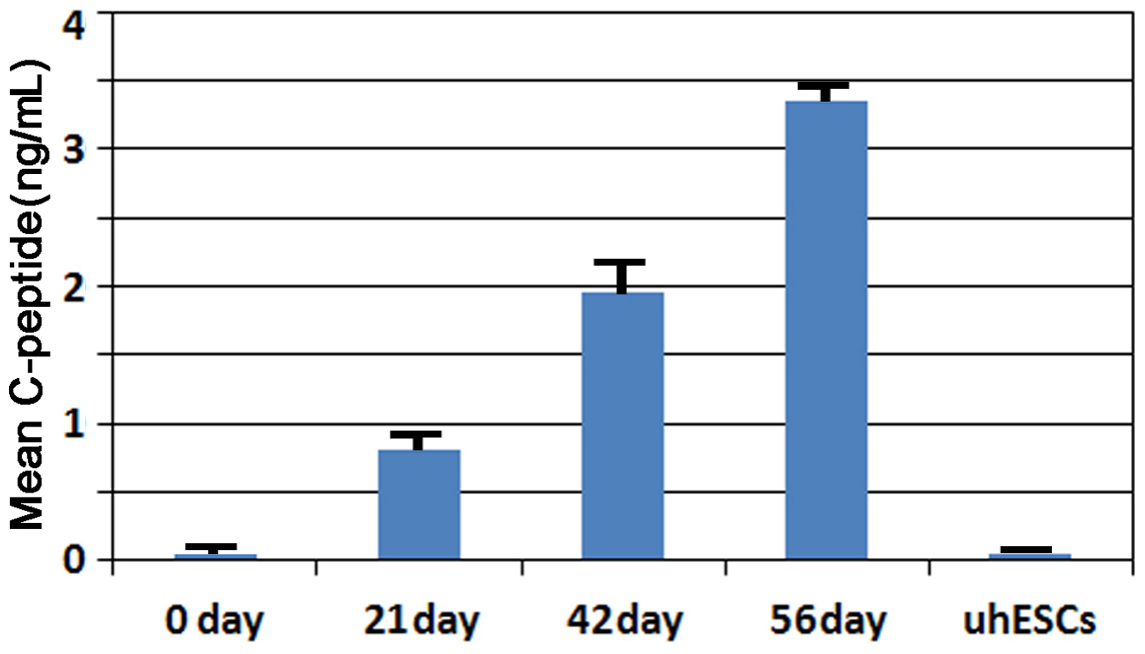
Human serum C-peptide levels on 21, 42 and 56 days following transplantation of differentiated cells and undifferentiated cells.

**Figure 8:**
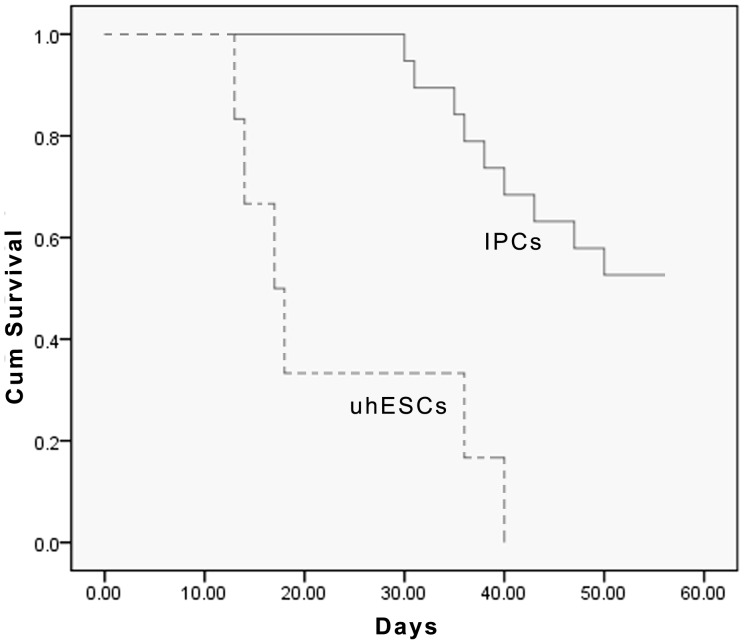
The Kaplan Meier survival curve comparing mice transplanted with hESC derived IPCs (n = 20) and undifferentiated hESCs (uhESCs, n = 6). *P_log rank_*<0.0001.

To further characterize the development of hESCs in vivo, about 5–7×10^6^ cells after stage 4 of differentiation were implanted into the epididymal fat pad in diabetic mice (Figure 5). Before implantation, the mean blood glucose level was 26.4±5.6 mmol/L. Three days after implantation, the blood glucose level decreased to 16.4±4.3 mmol/L. The blood glucose levels measured at 1–8 weeks after transplantation were 9.2±3.6, 8.2±3.2, 6.9±2.9, 7.0±3.2, 7.1±2.8, 7.6±2.6, 7.8±3.1, and 7.9±3.1 mmol/L, respectively (Figure 6). To assess the implanted cell survival, human serum C-peptide on 21, 42, and 56 days following transplantation of differentiated cells and undifferentiated cells (Figure 7). Of 20 mice transplanted with hESC-derived IPCs, six were found dead during 50 days follow-up period, and three were euthanized due to illness before or after 42 days post-transplant, respectively. The remaining 11 mice were euthanized for analysis at 56 days. As control group, 6 mice transplanted with undifferentiated hESCs, four mice were found dead in the course of the follow-up and two were euthanized due to illness. As compared to the IPCs group in which 56 days was the final euthanization point, none of these mice survived beyond 40 days. A Kaplan-Meier survival cure (Figure 8) using the log-rank test indicates a significant difference of P<0.0001 between the two groups of experimental mice. Notice that the KM curve for IPCs group is consistently higher than the KM curve for uhESCs group. These figures indicated that IPCs group has better survival prognosis than uhESCs group.

### Histological analysis of graft development in vivo

**Figure 9:**
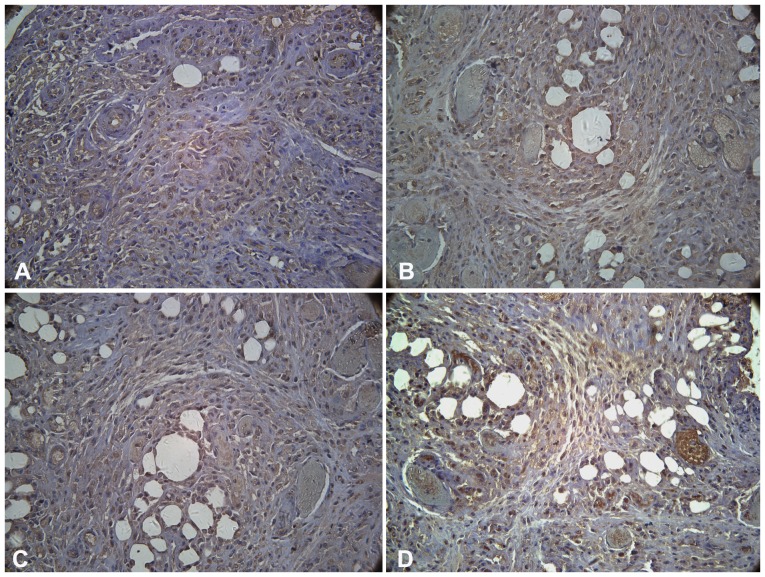
Photomicrographs of differentiated hESC-derived grafts at 8 weeks after transplantation. (A) human C-peptide, (B) GLUT-2, (C) PDX-1, and (D) glucagon. Scale bars: 400 µm.

In particular, tumor formation may be a problem associated with the graft of differentiated ESCs. While no terotomas or tumors were identified in these animals by gross or histological examination in our study. To assess the differentiation potential of the hESC-derived cells, we grafted the differentiated cells into the epididymal fat pads of 20 SCID/NOD mice. Aggregates of 5–7×10^6^ cells were deposited onto a Gelfoam sponge coated with Matrigel, and were then implanted into the epididymal fat pad in each mouse. Eight weeks after implantation, the grafts were removed and examined (Figure 9).

## Discussion

Fetal pancreatic development is an extremely complex process. In recent years, numerous research groups have generated insulin-producing cells in vitro using stepwise differentiation protocols that mimic the pancreatic development in vivo. To further optimize the pancreatic differentiation process, we compared the five-stage [7] and four-stage [8] induction protocols in preliminary studies. These studies revealed that the four-stage differentiation protocol, as used in the present study, is an optimized version of our previously reported method.

The five-stage induction protocol used to differentiate hESCs into pancreatic hormone-expressing endocrine cells is a sequential process involving the development of definitive endodermal cells, primitive gut tube cells, posterior foregut cells, pancreatic endodermal cells, endocrine precursor cells, and ultimately insulin-secreting cells. The insulin content of the terminally differentiated mature pancreatic cells was lower than that of primary adult human islet cells, and the differentiated cells had an equivalent function to that of the fetal pancreas. Moreover, the differentiated cells were not glucose responsive. Flow cytometry revealed that only 7.3% of cells derived from the five-stage protocol were positive for insulin [7]. Using differentiated hESCs via the four-stage induction protocol in this report, 17.1% of cells were positive for insulin, as determined by flow cytometry. The differentiated cells expressed insulin, glucagon, C-peptide, and GLUT-2, characteristic of mature endocrine cells. The differentiated hESCs also coexpressed C-peptide and PDX-1, a marker of mature islet cells. The hESC-derived cells were glucose-responsive and expressed C-peptide. When transplanted into the epididymal fat pads of SCID/NOD mice, the differentiated cells could reverse hyperglycemia for ≥8 weeks. Our results indicated that the hESC-derived mature pancreatic islet cells were functional in diabetic mice, and that the cells were functionally mature.

RT-PCR confirmed that the pancreatic progenitors derived from hESCs expressed of Gapd, Sox17 and Hnf4α, suggesting that the hESCs are of a pancreatic specification. Flow cytometry revealed that only 8.1% of the cells were positive for insulin and 2.0% were positive for C-peptide, indicating that the pancreatic precursors did not fully mature. Kroon et al [11] reported that transplantation of hESC-derived pancreatic endodermal cells into SCID/NOD mice gave rise to glucose-sensitive insulin-secreting cells and were capable of correcting hyperglycemia, suggesting that exposure to high glucose levels in vivo could enhance the maturation of pancreatic precursors. In this investigation, the differentiated hESCs expressed Pdx1, Insulin-1, Hnf4α, Glut2, and Nkx6-1, which suggests that the cells were functionally mature [12]. The co-expression of insulin, PDX1 and other key transcription factors, such as NKX6-1, is considered to be a specific functional characteristic of mature β cells [12], which has previously been reported only in islet β cells in vivo to our knowledge. Within 7 days of engraftment, blood glucose levels were significantly lower in diabetic mice that received the graft than in untreated diabetic controls. The differentiated hESCs also reverted hyperglycemia in the diabetic mice for ≥8 weeks, which indicates that the in vitro differentiation protocol could generate fully mature pancreatic islet cells.

EGF and its receptor are expressed throughout fetal pancreatic development, and the pancreas cannot develop following knockout of the EGF receptor. EGF is a key growth factor that stimulates the proliferation of fibroblasts, epithelial cells, and other cells. Co-administration of EGF and gastrin was reported to stimulate β cell regeneration in STZ-induced diabetic mice [13]. In our previous reports, we found that EGF can promote the differentiation of fetal bone marrow-derived mesenchymal stem cells [14] and fetal pancreatic stem cells [15] into islet cells.

In this study, EGF stimulated the proliferation of pancreatic progenitor cells. EGF and EGF receptor expression levels are lower in diabetic animals than in non-diabetic animals. EGF levels are also closely related to insulin secretion. Further research focusing on EGF and its receptor will reveal new options for cell replacement therapy for diabetes. Islet transplantation and stem cell transplantation represent new fields of replacement therapy for type 1 diabetes. However, the inadequate numbers of donor pancreases prevents wider application of such therapies [16]. Using xeno-islet isolation, the proliferation of IPCs in vitro and stem cell-derived pancreatic cell lines could make up for the shortage of donor islets. ESCs have an unlimited self-renewal capacity and the potential to differentiate into any cell type [17], [18], offering a new direction for the treatment of type 1 diabetes. Therefore, they retain some characteristics of tumor cells, including an unlimited proliferation potential, clonal growth, scarce inhibitory factors, and the ability to form teratomas. In this research, no teratoma were detected. However, the following are reasons that argue against this possibility: 1) The number of ES-implanted mice was limited; 2) Implantation time was short.We have completed bioinformatic studies of cancer-associated proteins [19] and have started studies to determine the associations of ESCs and tumors.

Stem cells (SCs) possess immunological and regenerative properties that could be harnessed to improve the treatment of type 1 diabetes (T1D). Furthermore, SC-derived insulin-producing cells are capable of engrafting and reversing hyperglycemia in mice [20]. Various stem cells have advantages and disadvantages. In addition to ESC-derived insulin-producing cells, congenic mesenchymal stem cells have been shown to reverse hyperglycemia in diabetic nonobese diabetic (NOD) mice [21].Cord blood stem cells(CB-SCs) have displayed immunodulatory and anti-inflammatory capabilities in vitro. CB-SCs expanded in vitro showed the lowest immunogenicity rate together with immunomodulatory effect [22].

In the future, we will conduct further screening studies to identify proteins associated with the oncogenicity of ESCs to understand the underlying mechanisms and perform comprehensive basic research before ESCs can enter preclinical trials. To ensure the efficacy and safety of transplantation, further studies should focus on the methods to generate IPCs comparable of natural β-cells phenotypically and functionally. Although cell replacement therapy for diabetes is still experimental and there is likely to be a long journey before ESCs can be used clinically for treating diabetes, stem cell transplantation represents a new direction of research for the treatment of diabetes, and has great prospects in terms of clinical application and academic research.

## References

[1] Shapiro AM, Lakey JR, Ryan EA, Korbutt GS, Toth E, et al (2000) Islet transplantation in seven patients with type 1 diabetes mellitus using a glucocorticoid-free immunosuppressive regimen. N Engl J Med 343: 230–238. 10911004 10.1056/NEJM200007273430401

[2] Donath MY, Halban PA (2004) Decreased beta-cell mass in diabetes: significance, mechanisms and therapeutic implications. Diabetologia 47: 581–589. 14767595 10.1007/s00125-004-1336-4

[3] Guo T, Hebrok M (2009) Stem cells to pancreatic beta-cells: new sources for diabetes cell therapy. Endocr Rev 30: 214–227. 19389995 10.1210/er.2009-0004 PMC2726841

[4] Jiang J, Au M, Lu K, Eshpeter A, Korbutt G, et al (2007) Generation of insulin-producing islet-like clusters from human embryonic stem cells. Stem Cells 25: 1940–1953. 17510217 10.1634/stemcells.2006-0761

[5] Keller G (2005) Embryonic stem cell differentiation: emergence of a new era in biology and medicine. Genes Dev 19: 1129–1155. 15905405 10.1101/gad.1303605

[6] Phillips BW, Hentze H, Rust WL, Chen QP, Chipperfield H, et al (2007) Directed differentiation of human embryonic stem cells into the pancreatic endocrine lineage. Stem Cells Dev 16: 561–578. 17784830 10.1089/scd.2007.0029

[7] D'Amour KA, Bang AG, Eliazer S, Kelly OG, Agulnick AD, et al (2006) Production of pancreatic hormone-expressing endocrine cells from human embryonic stem cells. Nat Biotechnol 24: 1392–1401. 17053790 10.1038/nbt1259

[8] Zhang D, Jiang W, Liu M, Sui X, Yin X, et al (2009) Highly efficient differentiation of human ES cells and iPS cells into mature pancreatic insulin-producing cells. Cell Res 19: 429–438. 19255591 10.1038/cr.2009.28

[9] Wang Y, Xu C, Wang H, Liu J, Hui S, et al (2012) Efficient derivation of human embryonic stem cell lines from discarded embryos through increases in the concentration of basic fibroblast growth factor. Hum Cell 25: 16–23. 22287285 10.1007/s13577-011-0039-7

[10] Korbutt GS, Elliott JF, Ao Z, Smith DK, Warnock GL, et al (1996) Large scale isolation, growth, and function of porcine neonatal islet cells. J Clin Invest 97: 2119–2129. 8621802 10.1172/JCI118649 PMC507287

[11] Kroon E, Martinson LA, Kadoya K, Bang AG, Kelly OG, et al (2008) Pancreatic endoderm derived from human embryonic stem cells generates glucose-responsive insulin-secreting cells in vitro. Nat Biotechnol 26: 443–452. 18288110 10.1038/nbt1393

[12] Murtaugh LC (2007) Pancreas and beta-cell development: from the actual to the possible. Development 134: 427–438. 17185316 10.1242/dev.02770

[13] Brand SJ, Tagerud S, Lambert P, Magil SG, Tatarkiewicz K, et al (2002) Pharmacological treatment of chronic diabetes by stimulating pancreatic beta-cell regeneration with systemic co-administration of EGF and gastrin. Pharmacol Toxicol 91: 414–420. 12688387 10.1034/j.1600-0773.2002.910621.x

[14] Hua XF, Wang W, Wang HY, Lian PW, Zhang SX, et al (2006) Differentiation of fetal mesenchymal stem cells into pancreatic islet-like clusters in vitro. Zhongguo Yishi Za Zhi 8: 1297–1299.

[15] Hua XF, Wang YW, Lian PW, Zhang SX, Li JY, et al (2012) Differentiation of fetal pancreatic stem cells into neuron-like and islet-like cells in vitro. Neural Regen Res 7: 506–510. 25745436 10.3969/j.issn.1673-5374.2012.07.005 PMC4348996

[16] Shapiro AM, Lakey JR, Ryan EA, Korbutt GS, Toth E, et al (2000) Islet transplantation in seven patients with type 1 diabetes mellitus using a glucocorticoid-free immunosuppressive regimen. N Engl J Med 343: 230–238. 10911004 10.1056/NEJM200007273430401

[17] Hoffman LM, Carpenter MK (2005) Characterization and culture of human embryonic stem cells. Nat Biotechnol 23: 699–708. 15940242 10.1038/nbt1102

[18] Liew CG, Moore H, Ruban L, Shah N, Cosgrove K, et al (2005) Human embryonic stem cells: possibilities for human cell transplantation. Ann Med 37: 521–532. 16278165 10.1080/07853890500379463

[19] Liu FJ, Hua XF, Wang WJ (2012) A new bioinformatics insight into human cancer-associated proteins. Oncol Rep 27: 1932–1936. 22426468 10.3892/or.2012.1714

[20] Fiorina P, Voltarelli J, Zavazava N (2011) Immunological application of stem cells in type 1 diabetes. Endocr Rev 32: 725–754. 21862682 10.1210/er.2011-0008 PMC3591677

[21] Jurewicz M, Yang S, Augello A, Godwin JG, Moore RF, et al (2010) Congenic mesenchymal stem cell therapy reverses hyperglycemia in experimental type 1 diabetes. Diabetes 59: 3139–3147. 20841611 10.2337/db10-0542 PMC2992776

[22] Francese R, Fiorina P (2010) Immunological and regenerative properties of cord blood stem cells. Clin Immunol 136: 309–322. 20447870 10.1016/j.clim.2010.04.010

